# Effect of Calcitonin Gene-Related Peptide on the Neurogenesis of Rat Adipose-Derived Stem Cells *In Vitro*

**DOI:** 10.1371/journal.pone.0086334

**Published:** 2014-01-21

**Authors:** Qin Yang, Xingli Du, Zhong Fang, Wei Xiong, Guanghui Li, Hui Liao, Jun Xiao, Guoping Wang, Feng Li

**Affiliations:** 1 Department of Orthopedics, Tongji Hospital, Tongji Medical College, Huazhong University of Science and Technology, Wuhan, Hubei, P R China; 2 Department of Pathology, Tongji Hospital, Tongji Medical College, Huazhong University of Science and Technology, Wuhan, Hubei, P R China; Baylor College of Medicine, United States of America

## Abstract

Calcitonin gene-related peptide (CGRP) promotes neuron recruitment and neurogenic activity. However, no evidence suggests that CGRP affects the ability of stem cells to differentiate toward neurogenesis. In this study, we genetically modified rat adipose-derived stem cells (ADSCs) with the CGRP gene (CGRP-ADSCs) and subsequently cultured in complete neural-induced medium. The formation of neurospheres, cellular morphology, and proliferative capacity of ADSCs were observed. In addition, the expression of the anti-apoptotic protein Bcl-2 and special markers of neural cells, such as Nestin, MAP2, RIP and GFAP, were evaluated using Western blot and immunocytochemistry analysis. The CGRP-ADSCs displayed a greater proliferation than un-transduced (ADSCs) and Vector-transduced (Vector-ADSCs) ADSCs (p<0.05), and lower rates of apoptosis, associated with the incremental expression of Bcl-2, were also observed for CGRP-ADSCs. Moreover, upon neural induction, CGRP-ADSCs formed markedly more and larger neurospheres and showed round cell bodies with more branching extensions contacted with neighboring cells widely. Furthermore, the expression levels of Nestin, MAP2, and RIP in CGRP-ADSCs were markedly increased, resulting in higher levels than the other groups (p<0.05); however, GFAP was distinctly undetectable until day 7, when slight GFAP expression was detected among all groups. Wnt signals, primarily Wnt 3a, Wnt 5a and β-catenin, regulate the neural differentiation of ADSCs, and CGRP gene expression apparently depends on canonical Wnt signals to promote the neurogenesis of ADSCs. Consequently, ADSCs genetically modified with CGRP exhibit stronger potential for differentiation and neurogenesis in vitro, potentially reflecting the usefulness of ADSCs as seed cells in therapeutic strategies for spinal cord injury.

## Introduction

Spinal cord injury (SCI) is a devastating neurological injury that often results in profound functional deficits and a frequent cause of mortality worldwide [1]–[3]. The pathophysiology of SCI is complicated, as this multifactorial and multiphasic event is determined not only by the initial mechanical insult but also by secondary processes, including ischemia [4]–[6], anoxia [7], free-radical formation [8], and excitotoxicity [9]; thus, various combination strategies, including the regeneration of neurons, neuroprotection from second injury, enhancement of axonal regrowth and synaptic plasticity, and inhibition of astrocytosis, are required for SCI repair.

Neural tissue engineering provides great promise for treating SCI and has achieved great success in experimental investigations [10], but the optimal cell donor remains unknown. For instance, embryonic stem cells (ESCs) can be induced to typical ectodermal cells in phenotype, but problems of histocompatibility, inadequate tissue supply, and ethical concerns exist [11], [12]. Neural stem cells (NSCs) were successfully used in neurogenesis in vitro and vivo [13], [14]; however, this process was obviously limited for clinical use reflecting an insufficient cell population harvested from neural tissue isolated from the brain of postmortem human cortices [15]. Similarly, bone marrow stromal cells (BMSCs) can be effectively differentiated into neurons and glial cells [16], [17], but bone narrow aspiration can harm patients, and problems of inadequate tissue supply are also observed.

As donor cells, adipose-derived stem cells (ADSCs) have shown many advantages, such as easy acquisition from sufficient adipose tissue, with a little harm to patients [18]–[21] and easier induction of differentiation and neurogenesis [22]–[25]. However, previous studies have indicated that the ability and capacity of ADSCs for neural differentiation are limited [23].

Calcitonin gene-related peptide (CGRP) is a neuropeptide found in nerves within the central and peripheral nervous systems. CGRP is primarily synthesized in the cell bodies of the dorsal root ganglion (DRG) and transported axonally to the peripheral and central endings of nerve fibers [26]. Moreover, CGRP has been recognized as a nerve regeneration-promoting peptide [27], and increasing CGRP expression could improve the survival of injured neurons and prevent neuronal loss. Furthermore, it has been suggested that CGRP might ameliorate SCI by inhibiting the release or production of TNF and increasing the expression of PGI2 [28]. Other studies have implicated CGRPs derived from spinal cord neurons in repair and regeneration after nerve injury [29]. Although numerous studies have characterized the stimulatory effects CGRPs on neurons, no studies have examined these effects on stem cells, particularly ADSCs.

In the present study, adult rat ADSCs were genetically modified to over-express CGRP, which would stimulate stem cells, facilitating neural differentiation and enhancing neurogenic capacity in vitro. Based on these results, we further speculate that CGRP-modified ADSCs might be effective seed cells in tissue engineering to promote the healing of SCI.

## Materials and Methods

Fetal bovine serum (FBS), trypsin, Dulbecco's modified Eagle's medium (DMEM) and Lipofectamine 2000 were purchased from Invitrogen, USA. PCR primers, Taq DNA polymerase, DNA ladder and oligo(dT)s were obtained from Sangon, China. The *Pme*I, *Pac*I, and *HindIII* restriction enzymes were purchased from NEB. The plasmid DNA extraction (Mini) kit was obtained from QIAGEN, UK. The *Escherichia coli* strain DH5a and the AdEasy Vector System were purchased from GeneChem, China. HEK293T cells (ATCC#: CRL-11268) were used to generate adenoviral particles. Sprague-Dawley rats were obtained from the Experimental Animal Center of Tongji Medical College and used in the following protocols approved through the Animal Care and Use Committee of Tongji Medical College of Huazhong University of Science and Technology (Permit Number: 20051007).

### Construction of plasmid vectors and adenoviral particles

The AdEasy Vector System was used to construct the pAd-EGFP adenoviral vector. This vector contained the EGFP reporter gene derived from pEGFP-C. The transfer vector pShuttle-CGRP was constructed using standard methods. pShuttle-CGRP was linearized with *Pme*I and co-transformed into the competent *E. coli* strain BJ5183 along with pAdeasy-1, the viral DNA plasmid. Briefly, 1 µg of the linearized recombinant transfer vector pShuttle-CGRP (5 µL) and 1.0 µL of the pAdEasy-1 vector (100 µg/µL) were added to 200 µL of competent-BJ5183 cells in a 14-mL culture tube. These components were gently mixed, incubated on ice for 1 h, heat-shocked at 42°C for 1 min and immediately returned to ice for 5 min. Subsequently, 1000 µL of LB media was added, and the cells were incubated with shaking (280 r/min) for 1 h at 37°C. The cells were plated onto 100-mm Petri dishes containing LB agar and incubated overnight at 37°C. The recombinant clones (pAd5-CGRP) were identified through restriction enzyme analysis.

pAdEasy-1 lacks E1 and E3, and the E1 function can be complemented in 293 cells. The recombinant adenoviral construct, pAd5-CGRP, was digested with *Pac*I to expose inverted terminal repeats and transfected into 293 cells to produce viral particles. The Ad5-CGRP construct was purified through two cesium chloride gradients, and the purified virus was desalted through dialysis at 4°C against 10 mmol/L Tris-HCl buffer containing 4% sucrose. The virus was stored in aliquots in liquid nitrogen, and the viral titer was determined using the Adeno-X™ Rapid Titer Kit.

### Isolation, culture and genetic modification of ADSCs

The isolation and cell culture of rats ADSCs were performed as previously described [23].To achieve high rates of viral infection, we used a protocol involving two centrifugation steps. The cells from sub-confluent cultures were harvested through treatment with 0.05% (w/v) EDTA in phosphate-buffered saline (PBS) containing MgCl_2_, CaCl_2_ and 0.25% (w/v) trypsin. The cells were seeded at a density of 100,000 cells/cm^2^ and centrifuged at 1000×g at 37°C for 10 min. The concentrated virus preparation was diluted 1∶1.5 with DMEM medium and applied to the pre-centrifuged cells, which were subsequently incubated at 37°C for 40 min, followed by a second centrifugation for 60 min. The infected cells were incubated under standard conditions overnight, followed by a medium change. To calculate the efficiency of infection in ADSCs was harvested and analyzed by flow cytometry to determine the proportion of cells expressing EGFP 24, 48, and 72 h after transduction. ADSCs without transduction and those transduced with Ad-EGFP-CGRP or Ad-EGFP are termed “ADSCs”, “CGRP-ADSCs”, and “Vector-ADSCs”, respectively. All experiments and cell number determinations were performed in triplicate.

### Fluorescence-activated cell sorting

FACS was carried out on a BD FACS (Aria Sorter, San Jose, CA) at 4°C and a pressure of 20 psi, using a laser at the 488 nm line, a 530/30 band pass filter, a 100 mm sorting tip, and a 34.2 kHz drive frequency, sterilized with 10% bleach. This instrument allowed us to characterize cells by size as well as fluorescence. Low flow rate improved the purity of cell sorting. Data acquisition and analyses were performed using BD FACS Diva 5.0.3 software, gated for a high level of EGFP expression. The clear separation of EGFP+ from EGFP- cells explains the ease of sorting. Sorted cells were re-analyzed to confirm that all were EGFP+. They were then plated on laminin-coated dishes.

### Formation of neurospheres from ADSCs

ADSCs cultured at high densities spontaneously formed spherical clumps of cells, isolated using 0.25% trypsin (Invitrogen, USA). We also collected the free-floating spheres released from the cell culture surface into the culture media. The spheres of cells were transferred to a Petri dish and cultured in Neurobasal medium (Invitrogen, USA) supplemented with B27 (Invitrogen, USA), 20 ng/ml of bFGF, and 20 ng/ml of EGF (Sigma, St. Louis, MO, USA) for 4–7 days. The culture density of the spheroid bodies was maintained at 10–20 cells/cm^2^ to prevent self-aggregation.

### In vitro differentiation of ADSCs to neural cells

For neural lineage differentiation, neurospheres derived from ADSCs were layered onto PDL-laminin double-coated chamber slides. The spheres were cultured and maintained for 10 days in NB media containing only the B27 supplement. Approximately 70% of the media was replaced every 4 days. These cells were examined at 1, 3 and 7 days after differentiation using a western blot analysis. All data represent at least three different experiments.

### Morphology, growth curve of ADSCs after transduction

After transduction for 3 and 7 days, the cells of each group were plated at a cell density of 2×10^4^/ml, and the cell morphology was observed under an inverted microscope. In addition, the growth curve of the two groups was plotted using an MTT assay.

### Cell apoptosis assay

At the indicated times, cells were harvested using trypsin/EDTA, counted, and collected through centrifugation in PBS. Phosphatidylserine (PS) exposure on the outer leaflet of the plasma membrane was detected using the fluorescent dye Annexin V-FITC Apoptosis Detection Kit (BD Biosciences, USA) according to the manufacturer's instructions. All data were collected and analyzed using Lysis II software (BD Biosciences, USA). The experiments were repeated 3 times and the results are presented as the means ± SD.

### Western blot analysis

The cells were washed twice with ice-cold phosphate-buffered saline (PBS) and directly lysed in Laemmli buffer. The lysate was sonicated, boiled for 5 min and centrifuged at 16,000×g for 10 min at 4°C. The supernatant was recovered as total cell lysate, aliquoted and stored at −80°C. Equal amounts of protein (10 µg) were separated through 8% SDS-PAGE and electro-transferred onto 0.45 µm polyvinylidene difluoride membranes (Millipore, Bedford, USA). Following transfer, the membranes were blocked with a solution of 0.1% Tween 20/TBS (TBS/T) containing 5% non-fat milk for 1 h at room temperature and subsequently incubated overnight at 4°C with monoclonal mouse anti-human CGRP (Santa Cruz Biotechnology, Santa Cruz, CA, USA, final dilution 1∶400), Bcl-2 (GeneTex, USA, final dilution 1∶300), Wnt-1, Wnt-3a, Wnt-5a, Wnt-7 and β-catenin (Sigma, St. Louis, MO, USA, final dilution 1∶400) antibodies or rabbit polyclonal anti-human nestin, MAP2, RIP, and GFAP antibodies (Sigma, St. Louis, MO, USA, final dilution 1∶500). The bands were visualized using nitroblue tetrazolium/5-bromo-4-chloro-3-indolyl-phosphate. GAPDH served as an endogenous control. For densitometric analyses, the blots were scanned and quantified using Quantity One analysis software (Bio- Rad, Hercules, CA, USA). The results were expressed as a percentage of GAPDH immunoreactivity.

### Immunocytochemistry analysis

For analysis of neural differentiation of ADSC, differentiated cells were fixed with 4% paraformaldehyde, and incubated with 10% goat serum to prevent nonspecific antibody binding. The cells were incubated overnight at 4°C with several rabbit polyclonal anti-human Nestin, MAP2, RIP, and GFAP antibodies (Sigma, St. Louis, MO, USA, final dilution 1∶500). After extensively washing in PBS, the cells were then incubated for 30 min with Alexa fluor 488 conjugated secondary antibodies (1∶400–500; Invitrogen, Carlsbad, CA, USA). Controls in which primary antibodies were omitted or replaced with irrelevant IgG resulted in no detectable staining. Specimens were examined using a Leica TCS SP2 laser scanning microscope equipped with three lasers (Leica Microsystems, USA). Immunocytochemical studies were repeated at least three times.

### Statistical analysis

Each experiment was repeated three times. All data are represented as the mean ± SD, and the statistical analysis was performed using the SPSS software package (Version 12.0). The data were analyzed using the independent-samples t-test and a paired t-test. The CGRP data were not normally distributed and were therefore tested using the Wilcoxon signed-rank test. p<0.05 was considered statistically significant.

## Results

### Over-expression of CGRP in genetically modified ADSCs

**Figure 1:**
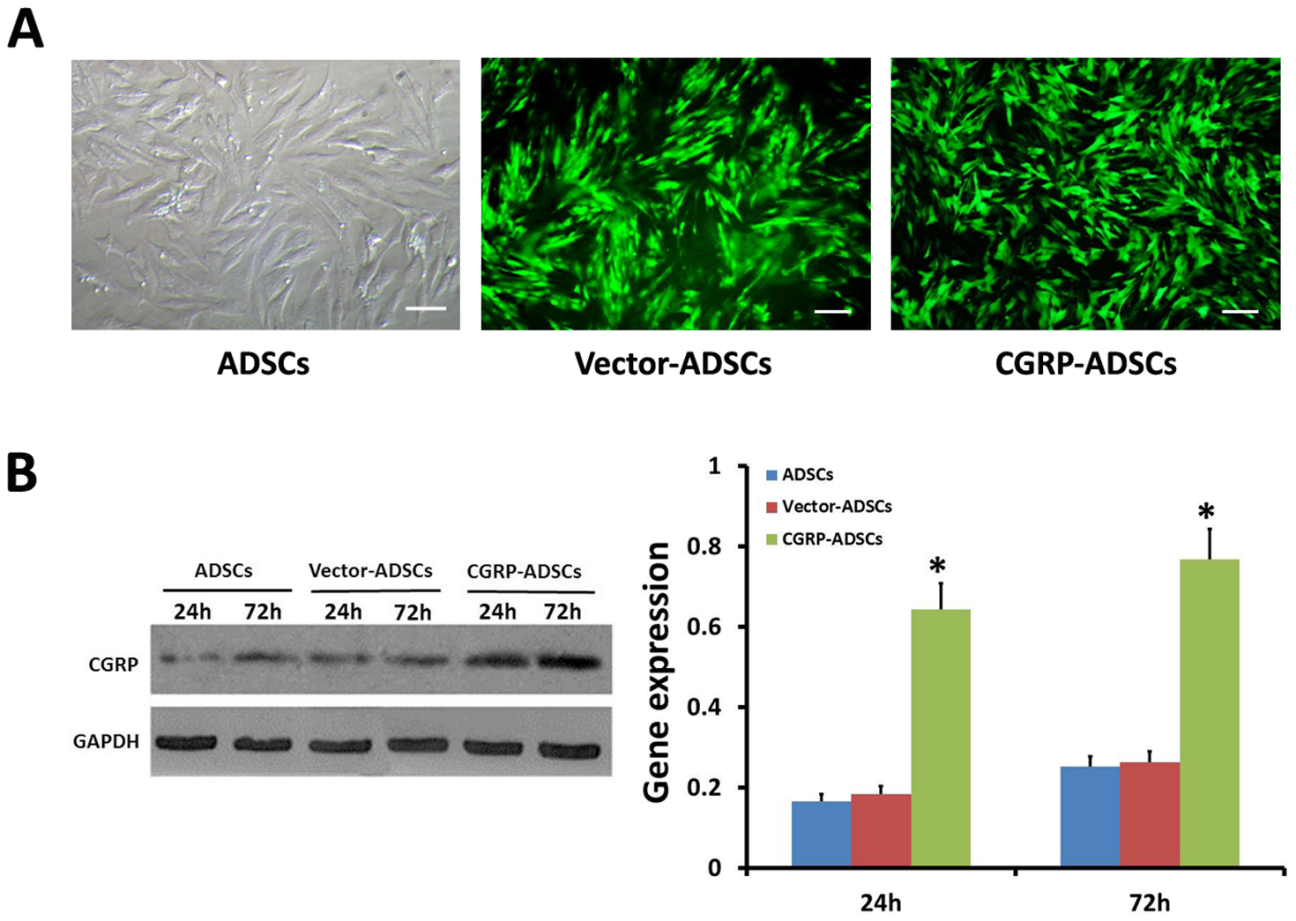
Overexpression of CGRP in genetically modified ADSCs. (A) The transduction efficiency was determined using flow cytometry at 72 h after transduction. Scale bar equals 100 µm. (B) The expression of CGRP among all groups was detected on days 1 and 3. *p<0.05, CGRP-ADSCs group vs. controls.

The transduction efficiency for genetically modified ADSCs was evaluated according to the expression of EGFP gene using flow cytometry. At 72 h, the transduction efficiency peaked, showing approximately 81.5% (**Fig. 1A**) Vector-ADSCs or CGRP-ADSCs. To accurately evaluate the expression of CGRP among all the groups, a western blot analysis was performed on days 1 and 3. As shown in **Fig. 1B**, significantly higher CGRP expression in CGRP-ADSCs was observed on either days 1 or 3 (*p*<0.05), compared with ADSCs and Vector-ADSCs.

### Morphology and cell growth characterization of ADSCs after transduced CGRP gene

**Figure 2:**
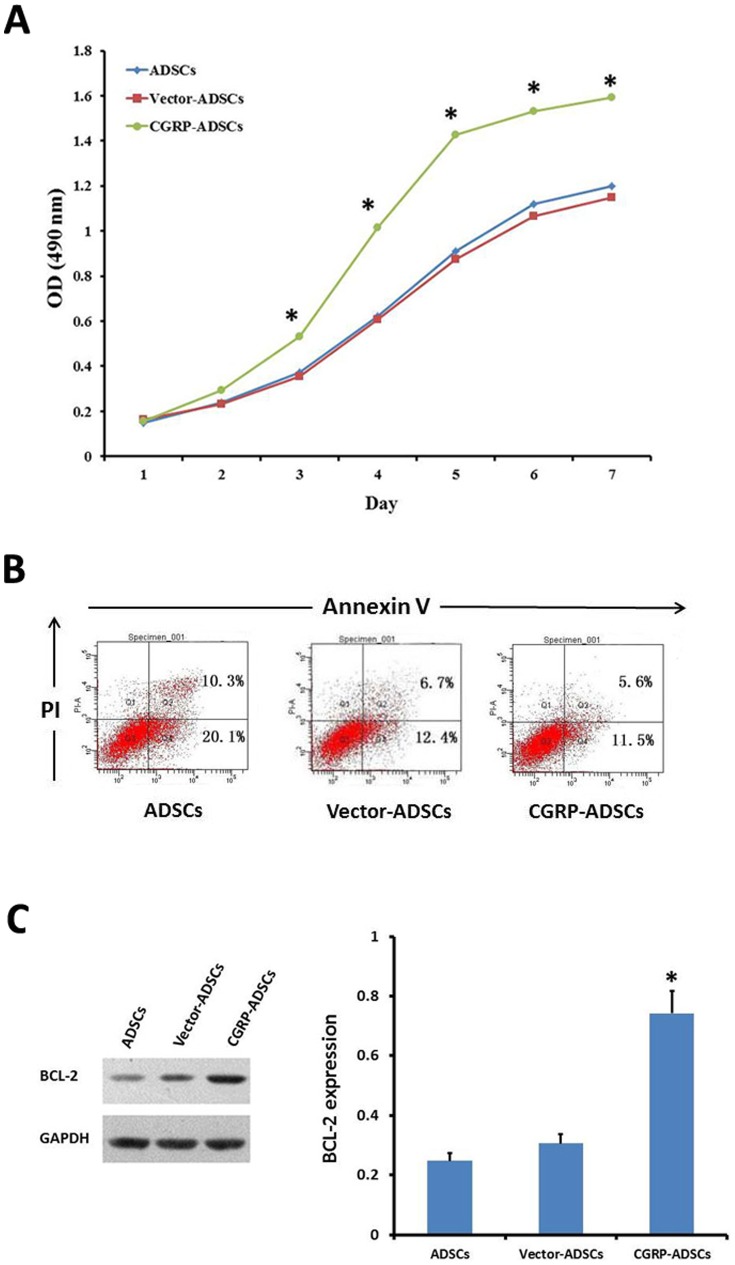
CGRP overexpression altered the characterization of ADSCs. (A) The growth curves showed that CGRP-ADSCs had a significantly higher proliferation than ADSCs or V-ADSCs. *p<0.05, CGRP-ADSCs group vs. controls. (B) Apoptosis was quantified through FACS analysis after staining with Annexin V and PI. The Annexin V^+^/PI^−^ cells appeared early in the apoptotic process. The viable cells were Annexin V^−^/PI^−^. The quantitative analysis showed that the number of TUNEL-positive cells in CGRP-ADSCs was significantly decreased compared with ADSCs and V-ADSCs. (**C**) The expression of BCL-2 among all groups was detected on day 3. *p<0.05, CGRP-ADSCs group vs. controls.

The ADSCs genetically modified with CGRP exhibited bright green EGFP fluorescence. Despite of some colony growth, the CGRP-ADSCs were evenly distributed and the cell morphology predominantly showed a heterogeneous population of long, spindle-shaped cells. Conversely, ADSCs or Vector-transduced ADSCs primarily grew in a monolayer style as flat fibroblast-like cells. Meanwhile, the proliferative capacity of each group was calculated using an MTT assay, and the results were differently displayed on growth curves (**Fig. 2A**). Apparently, the proliferation of CGRP-ADSCs was significantly higher than the other groups (*p*<0.05).

### CGRP modified ADSCs protect against apoptosis *in vitro*

To examine the capability of CGRP-ADSCs to protect against apoptosis, the rates of cell apoptosis were assessed through Flow Cell detection. The rates of cell apoptosis in ADSCS and Vector-ADSCs significantly exceeded that of CGRP-ADSCs by almost 1.7-fold according to the detection of Annexin V/PI staining (**Fig. 2B**). In addition, the quantitative analysis showed that the expression of BCL-2 in CGRP-ADSCs was significantly higher than that in the other groups on day 3 (*p*<0.05) after transduction (**Fig. 2C**). These findings demonstrated that the CGRP-modified ADSCs protect against apoptosis *in vitro*.

### Neurosphere formation and morphological changes of CGRP-ADSCs on neural induction

**Figure 3:**
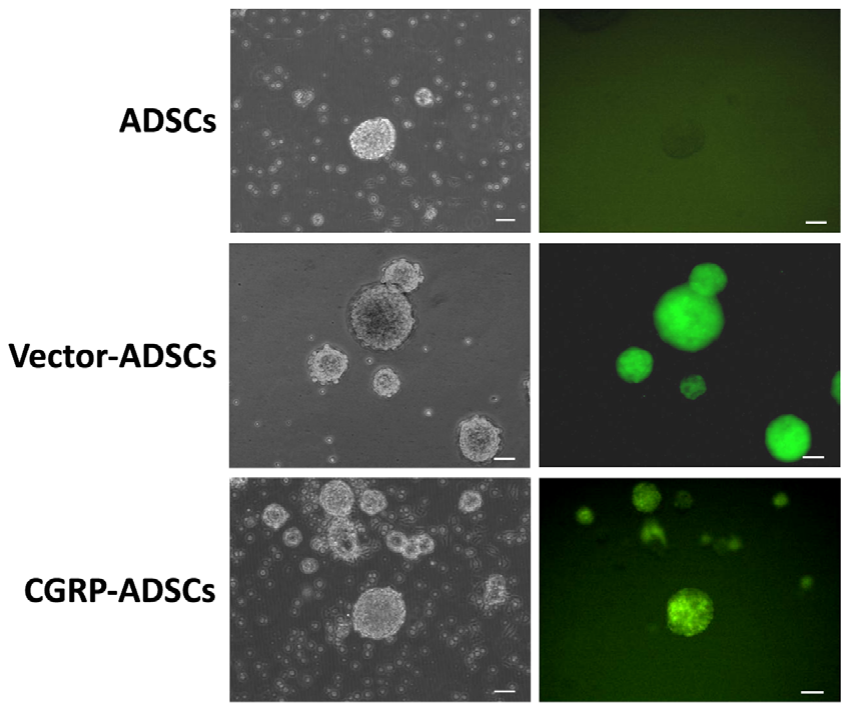
CGRP overexpression promoted neurosphere formation. When ADSCs approached densities of approximately 80%, all groups were induced toward the neurogenic lineage through neurosphere formation. Apparently, the size and quantity of neurospheres were increased CGRP-ADSCs compared with the other groups. Scale bars = 100 µm.

When the ADSCs approached densities of approximately 80%, all groups were induced toward neural differentiation. First, neurospheres were formed as shown in **Fig. 3**, and the size and quantity of neurospheres on CGRP-ADSCs were increased compared with the other groups.

**Figure 4:**
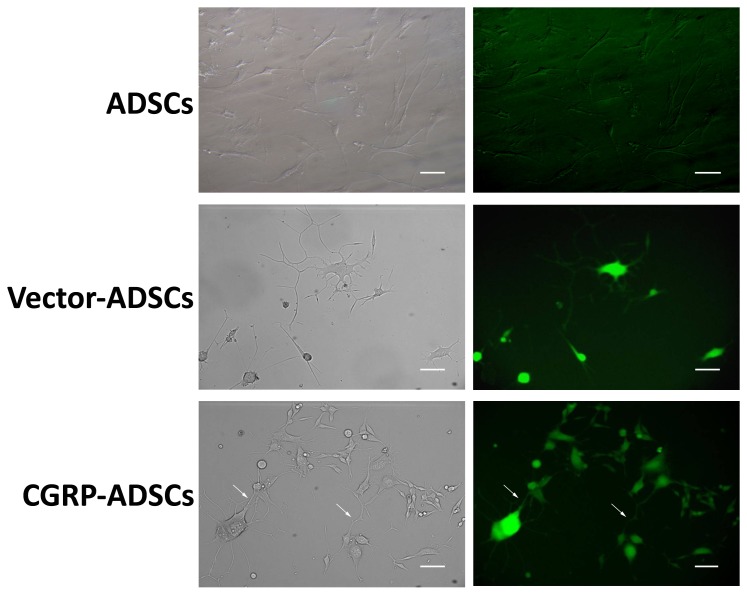
Effect of CGRP on morphological changes of ADSCs on neural induction. On light microscopy or fluorescence microscopy on day 3 after neural induction, ADSCs develop characteristic round cell bodies with several branching extensions on day 3 after neural induction; Specially, the CGRP-ADSCs exhibited more branching extensions on contacting with neighboring cells widely (as show on white arrows). Scale bars = 50 µm.

Subsequently, the morphology of some single cells, particularly CGRP-ADSCs, began to change and developed into characteristic round cell bodies with several branching extensions as shown in Fig. 4, concomitantly expressing EGFP fluorescence. Approximately 60% of the CGRP-ADSCs were bipolar or multipolar in shape and more of these cells contacted neighboring cells widely.

### Neural markers expression in differentiated ADSCs

**Figure 5:**
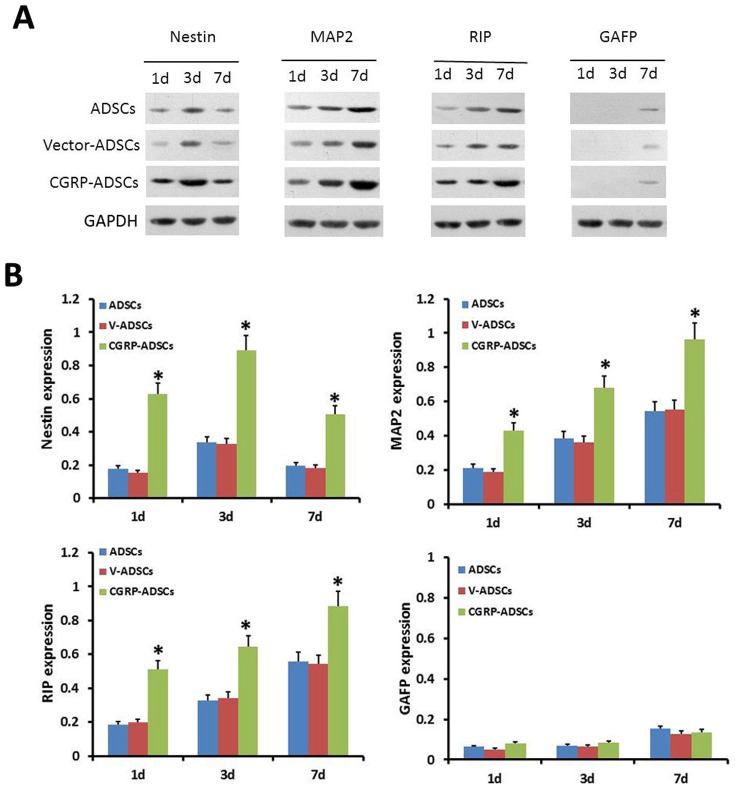
Results of neural markers expressions by Western blot. (A), (B) Western blot analysis to examine the expression of various protein markers after neural induction on 1, 3, and 7 days. *p<0.05, CGRP-ADSCs group vs. controls.

**Figure 6:**
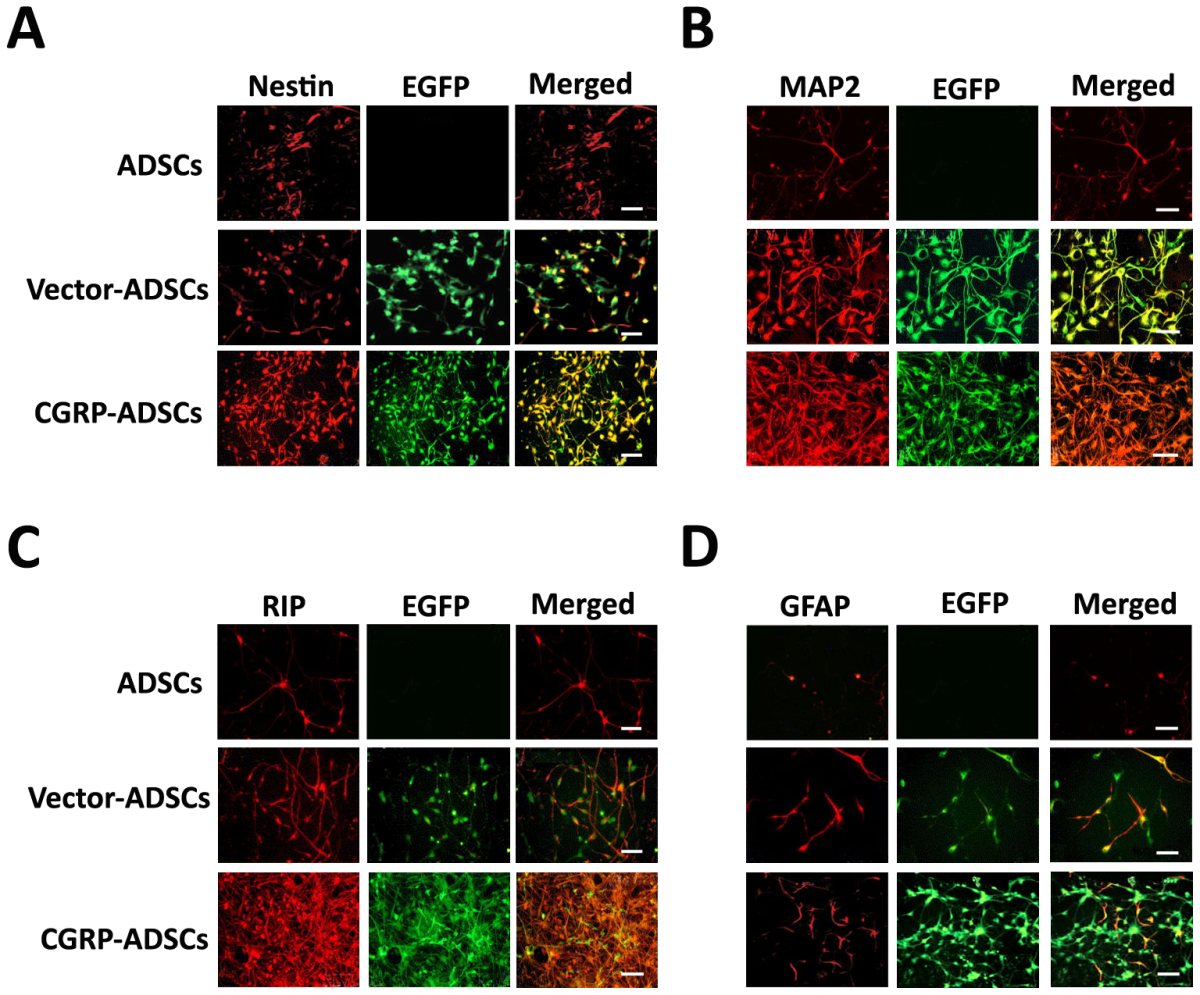
Results of Neural markers expressions by immunostaining. Immuostaining analysis to detect the expressions of various protein markers after neural induction on 7 days. (A) expression of Nestin (red); (B) expression of MAP2 (red); (C) expression of RIP (red); and (D) expression of GAFP (red). Scale bars = 100 µm.

To fully characterize the differentiated ADSCs after neural induction, western blot analyses for specific antigens indicative of neural cell lineages were performed on days 1, 3 and 7. The expression of Nestin in CGRP-ADSCs early after induction, particularly on day 3, indicated a high degree of neural differentiation. However, after 7 days of induction, the rate of differentiation was remarkably decreased (**Fig. 5A**). Despite the similar trend in ADSCs or Vector-ADSCs, the expression of Nestin in CGRP-ADSCs showed significantly higher level compared with the other groups on days 1, 3 or 7 (*p*<0.05) (**Fig. 5B** **;** **Fig. 6A**). The expression of MAP2 and RIP apparently showed an up-regulated expression profile at the whole phases of neural-induced commitment among all groups (**Fig. 5A**), but the expression of these proteins in CGRP-ADSCs was significantly higher than that in the other groups on days 1, 3 or 7 (*p*<0.05) (**Fig. 5B** **;** **Fig. 6B** **;** **Fig. 6C**). Lower levels of GFAP expression among all groups were confirmed on days 1, 3 or 7. Moreover, there was no significant difference in CGRP-ADSCs (*p*>0.05), compared with the other groups (**Fig. 5A** **and** **Fig. 5B** **;** **Fig. 6D**).

### Expression of Wnt signal proteins as a neural indication

**Figure 7:**
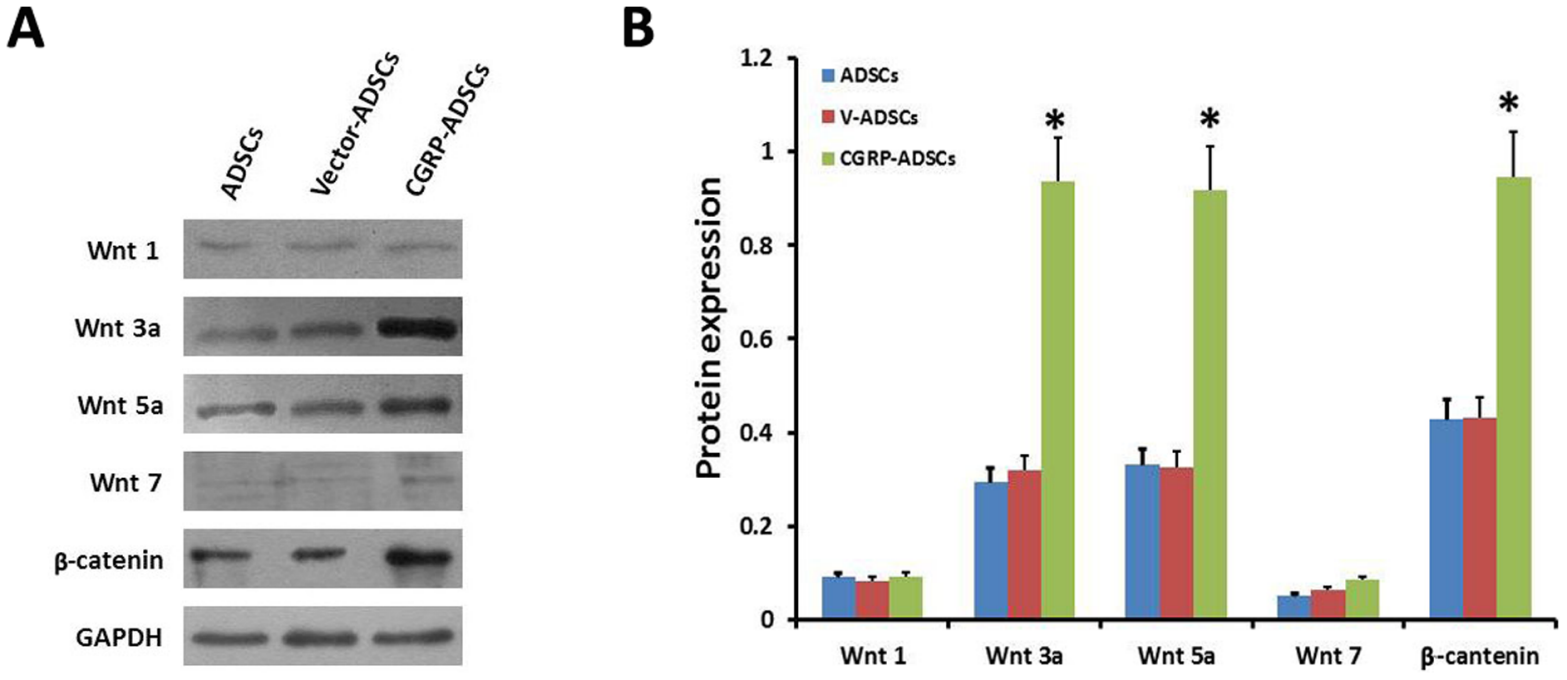
Expression of Wnt signal proteins on neural differentiation of ADSCs. (A), (B) During neurogenesis, Wnt 3a, Wnt 5a and β-catenin expression was detected among all groups. Moreover, in the expression of these signals in CGRP-ADSCs was significantly higher than that in the other groups (*p*<0.05). However, the expression of Wnt 1 and Wnt 7 among all groups was detected at lower levels in all groups, and there was no significant difference among the groups.

To fully characterize the regulation of the neural differentiation of ADSCs, western blot analyses for specific antigens indicative of Wnt/β-catenin signaling were performed on induction day 7. The data from these analyses indicated a high degree of Wnt 3a, Wnt 5a and β-catenin expression among all groups (**Fig. 7A**). Moreover, the CGRP-ADSCs showed significantly higher expression of these neural markers compared with the other groups (p<0.05) (**Fig. 7B**). However, the expression of Wnt 1 and Wnt 7 was low among all groups (**Fig. 7A**), and no significant difference was observed among the groups (**Fig. 7B**).

## Discussion

Genetically modified neural tissue engineering is an attractive approach with great potential for use in the treatment of spinal cord injury or brain damage [30]. Many studies have focused on bone marrow mesenchymal or neural stem cells. However, few related reports on adipose tissue-derived stem cells are available [31]. Adipose tissue has several advantages, including abundance and ease of acquisition [18]–[21] and easier induction to different lineages [22]–[25], and this tissue is becoming a promising seed cell source [23]. In addition, adenoviral vectors transduce both dividing and non-dividing cells and incorporate into the host genome, facilitating prolonged target gene expression, high transfection efficiency, and low toxicity [32], [33]. In our study, ADSCs were selected as donor cells, and adenoviral vectors were used for transduction. CGRP-transduced ADSCs could be transduced with high transduction efficiency, (approximately 81.5%), demonstrating that the transduction of ADSCs using an adenoviral vector was a feasible and efficient method to incorporate a foreign gene. Moreover, on days 1 and 3 after transduction, the over-expression of CGRP was detected at a significantly higher level than that in the other control groups (*p*<0.05). Consequently, these results demonstrated that ADSCs and adenovirus-mediated gene-targeting vectors were applicable to tissue engineering.

The calcitonin family of peptides has been extensively studied in neural system disorders over the past few years because of the effects of these peptides on neural cells, particularly neurons. However, little is known about the effect of CGRPs on the proliferation or differentiation of stem cells. In this study, it was the over-expression of CGRP in CGRP-ADSCs promoted cell proliferation and significantly higher growth rate (p<0.05) compared with the control groups. Furthermore, the apoptotic cell number in CGRP-ADSCs was remarkably reduced after the CGRP genetic modification. Meanwhile, the expression of BCL-2 in CGRP-ADSCs was significantly higher than that in the other groups on day 3 (p<0.05). Hence, CGRP over-expression should reduce death and apoptosis of ADSCs, and these cells also retain their proliferation capacity and likely generate different lineages, particularly neurogenesis. Previous studies have shown that CGRP acts as a survival factor, inhibiting apoptosis in liver cells [34] [35]. The anti-apoptotic action of CGRP is potentially regulated, in part, through the ERK signaling pathway [36], further increasing our understanding of the biological mechanism of CGRP.

Thus, the results of the present study revealed that CGRP-ADSCs undergo morphological and phenotypical changes consistent with neural differentiation. First, neurospheres were formed, followed by CGRP-ADSCs aggregation after neural induction. Furthermore, the size and quantity of the neurospheres in CGRP-ADSCs were increased compared with the other groups. Second, the morphology of CGRP-ADSCs developed into characteristic round cell bodies, with more branching extensions, bipolar or multipolar in shape, and some ADSCs contacted neighboring cells widely (Fig. 4). Third, specific antigens indicative of neural cell lineages were detected after neural induction. The expression of Nestin, typically observed at a high level in neural stem cells, representing potential neurogenic capacity [37], exhibited a high degree in CGRP-ADSCs early after induction. However, at 7 days after induction, the high levels of Nestin expression were remarkably reduced. The phenomenon of high Nestin expression is consistent with our previous work [23] and other studies in stromal cells of bone marrow origin [38], [39], demonstrating that ADSCs with or without Ad-CGRP transduction might retain a native potential for neural differentiation. The strong up-regulated expression of MAP2, as a neuron marker [40], and RIP, as an oligodendrocyte marker [41], in neural induction were observed in CGRP-ADSCs on days 1, 3 or 7. Moreover, the expression of these markers was significantly higher than that observed in the other groups (p<0.05). Conversely, the expression of GFAP, as a marker for astrocytes [42], was nearly undetectable until day 7, showing slight expression among all groups. Taken together, these results demonstrated that ADSCs, with or without genetic modification through CGRP, could promote differentiation into neurocytes rather than astrocytes, and CGRP-ADSCs showed easier neurogenesis than ADSCs or Vector-ADSCs under the conditions provided in this study.

In addition, Wnt/β-catenin signaling was detected when ADSCs were induced to neural differentiation in this study. On day 7 of neural induction, the increased expression of the canonical Wnt signals, Wnt 3a, Wnt 5a and β-catenin, was observed among all groups. Furthermore, significantly higher expression of these markers was observed in CGRP-ADSCs compared with the other groups (*p*<0.05). However, lower levels of Wnt 1 and Wnt 7 expression were detected, showing no significant difference among the groups. Based on these results, it is reasonable to speculate that canonical Wnt signals, primarily Wnt 3a, Wnt 5a and β-catenin, are involved in the regulation of the neural differentiation of ADSCs, suggesting that the CGRP gene could up-regulate the expression of canonical Wnt signals during neurogenesis in ADSCs [43]. However, additional research is needed to characterize the mechanisms of molecular regulation in detail.

In summary, this study demonstrated that the adenovirus-mediated CGRP-ADSCs successfully underwent neurogenesis *in vitro*, maintained a high proliferative capacity and successfully secreted extracellular matrix. CGRP-ADSCs might also serve as ideal seed cells for neural tissue engineering. Whether the CGRP-ADSCs retain the same ability to differentiate into neurogenic lineages and repair SCI *in vivo* should be further tested.
